# Streamlined quantitative BOLD for detecting visual stimulus-induced changes in oxygen extraction fraction in healthy participants: toward clinical application in human glioma

**DOI:** 10.1007/s10334-023-01110-1

**Published:** 2023-08-09

**Authors:** Fatemeh Arzanforoosh, Avery J. L. Berman, Marion Smits, Esther A. H. Warnert

**Affiliations:** 1 Department of Radiology and Nuclear Medicine, Erasmus MC, Rotterdam, The Netherlands; 2 Department of Physics, Carleton University, Ottawa, ON Canada; 3 University of Ottawa Institute of Mental Health Research, Ottawa, ON Canada; 4 Medical Delta, Delft, The Netherlands

**Keywords:** Magnetic resonance imaging, Brain oxygenation, Visual stimulation, Healthy volunteers, Glioma

## Abstract

**Objective:**

Monitoring brain oxygenation is critical in brain tumors, as low oxygenation influences tumor growth, pathological angiogenesis, and treatment resistance. This study examined the ability of the streamlined quantitative (sq)BOLD MRI technique to detect oxygenation changes in healthy individuals, as well as its potential application in a clinical setting.

**Methods:**

We used the asymmetric spin echo (ASE) technique with FLAIR preparation, along with model-based Bayesian inference to quantify the reversible transverse relaxation rate (*R*_2_^'^) and oxygen extraction fraction (OEF) across the brain at baseline and during visual stimulation in eight healthy participants at 3T; and two patients with glioma at rest only.

**Results:**

Comparing sqBOLD-derived parameters between baseline and visual stimulation revealed a decrease in OEF from 0.56 ± 0.09 at baseline to 0.54 ± 0.07 at the activated state (*p* = 0.04, paired *t* test) within a functional localizer-defined volume of interest, and a decline in *R*_2_^'^ from 6.5 ± 1.3s^−1^ at baseline to 6.2 ± 1.4s^−1^ at the activated state (*p* = 0.006, paired *t* test) in the visual cortex.

**Conclusion:**

The sqBOLD technique is sensitive enough to detect and quantify changes in oxygenation in the healthy brain and shows potential for integration into clinical settings to provide valuable information on oxygenation in glioma.

**Supplementary Information:**

The online version contains supplementary material available at 10.1007/s10334-023-01110-1.

## Introduction

The brain depends on a continuous and adequate supply of oxygen to maintain its functional integrity. Chronically impaired delivery of oxygen to brain cells results in hypoxic injury and neurodegeneration [1]. Low tumor oxygenation constitutes one of the major concerns for patients with brain tumors, as it plays a central role in tumor development, angiogenesis, and resistance to treatment, and is associated with a poorer prognosis [2]. However, despite the significance of measuring blood oxygenation and advances in MRI techniques in this field, translating these techniques into clinical practice and validating them in disease states remains challenging.

Quantitative blood oxygen level-dependent (qBOLD) techniques have garnered attention in the field of magnetic resonance imaging (MRI) since their inception, as they offer a method for quantifying the oxygen extraction fraction (OEF) rather than solely providing qualitative assessments of blood oxygenation changes [3], as is the case with traditional BOLD imaging. OEF, which is the ratio of oxygen extracted from blood by tissue to the total oxygen content of arterial blood, is calculated using a biophysical model that relates both OEF and the deoxygenated blood volume (DBV) to the reversible transverse relaxation rate (*R*_2_^'^) [3]. *R*_2_^'^ is sensitive to magnetic field inhomogeneities, often arising from differences in tissue susceptibilities. Deoxygenated blood causes such magnetic field inhomogeneities because it is paramagnetic; the iron centers of deoxygenated hemoglobin molecules have unpaired electrons that perturb the magnetic field. In contrast, oxygenated hemoglobin is diamagnetic because all electrons become paired during binding of oxygen molecules [4]. This makes *R*_2_^'^ a versatile tool, influenced by various factors linked to changes in blood oxygenation, such as neural activity and pathophysiological conditions [5–7]. DBV, on the other hand, represents the volume of partially deoxygenated blood per unit volume or mass of tissue.

qBOLD techniques can be classified into two primary groups based on their measurement strategies. The first group includes techniques that allow for the direct measurement of *R*_2_^'^, and frequently DBV, such as GESFIDE (Gradient Echo Sampling of FID and Echo), GESSE (Gradient Echo Sampling of Spin Echo), and ASE (Asymmetric Spin Echo) [5]. The second group comprised multi-parametric qBOLD (mqBOLD) approaches that use separate measurements of *R*_2_ (irreversible transverse relaxation rate) and effective transverse relaxation rate (*R*_2_^'^) to measure *R*_2_^'^ as well as DBV to estimate brain tissue oxygenation [8]. Streamlined-qBOLD (sqBOLD) is a new *R*_2_^'^-based technique that employs an ASE sequence along with a preparation pulse to reduce contamination from the interstitial or cerebrospinal fluid (CSF) compartment using fluid-attenuated inversion recovery (FLAIR-ASE) [9]. The technique offers the advantage of minimizing *R*_2_^'^ bias caused by CSF, as well as a simpler *R*_2_^'^ analysis process compared to other mentioned qBOLD methods since *R*_2_-weighting is constant throughout [5].

sqBOLD has been successfully applied in healthy participants and patients with ischemic stroke [9, 10]. However, the sensitivity of this technique to different levels of OEF in the healthy brain and in the complex microvascular environment of neoplasms is yet unclear. One way of assessing such sensitivity in the healthy brain is by manipulating OEF through a functional task, e.g., visual stimulation. During a functional task, increased neural activity results in a hemodynamic response that increases local cerebral blood flow (CBF) beyond the increase in metabolic demand, leading to a decrease in the ratio of extracted oxygen to delivered blood and a subsequent decrease in measured OEF [11].

On the other hand, currently, there is only very limited research exploring the use of qBOLD for measuring OEF in human brain tumors, which has impeded the integration of these techniques into clinical practice [12, 13]. These studies employed the mqBOLD technique, which requires separate measurements of DBV as well as *R*_2_ and *R*_2_^*^ to calculate *R*_2_^'^. Findings from these studies suggest that high-grade tumors exhibit higher OEF than lower-grade ones, and the volume of high OEF increases with tumor tissue malignancy.

This study is guided by two primary objectives. First, we sought to investigate the sensitivity of sqBOLD to regional changes in OEF. To optimize this, we initially examined the effect of echo time (TE) on our local implementation of the technique and evaluated its impact on the sqBOLD-derived parameters, *R*_2_^'^, DBV, and OEF. Subsequently, we assessed the sensitivity of sqBOLD in detecting task-related OEF changes in healthy participants during visual stimulation. Second, we applied this technique to measure *R*_2_^'^, DBV, and OEF at rest in two patients with primary brain tumors (gliomas)—one with a low-grade and the other with a high-grade tumor—illustrating the feasibility of using this technique in a clinical context to measure regional variations in oxygen-related parameters for these patients.

## Materials and methods

### Participants

Ten healthy volunteers (7 males; mean age 26 ± 6 years) and two patients with glioma (1 male and 1 female; ages 74 and 75 years) were recruited for the study. All healthy participants underwent MRI screening to exclude cerebrovascular and neurological diseases. The patients were diagnosed with low-grade glioma (astrocytoma, IDH-mutant-type grade 2) and high-grade glioma (glioblastoma IDH-wildtype grade 4), according to the World Health Organization 2021 criteria [14]. However, during the analysis phase, two participants were post-hoc excluded—one due to incomplete fMRI data and another due to incomplete sqBOLD data with visual stimulation. This resulted in the final sample size of eight that is reported in this study. Written informed consent was obtained from both healthy participants and patients for storing and using their data for research, both prospectively and retrospectively. The study was approved by the local ethics board and conducted in accordance with the Declaration of Helsinki.

### Magnetic resonance imaging

**Fig. 1:**
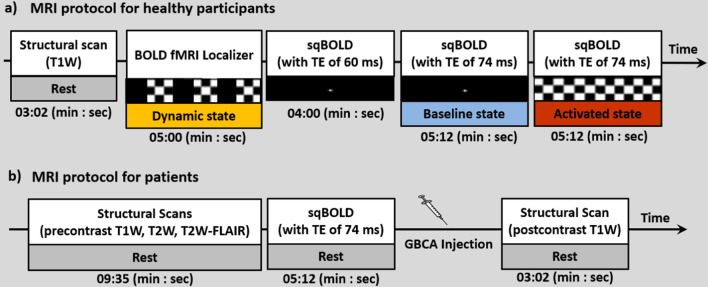
A schematic representation of the protocol design for **a** healthy participants and **b** patients. **a** In the healthy participant protocol, a T1-weighted (T1W) scan was acquired, followed by a BOLD fMRI localizer run, during which participants viewed a flickering checkerboard with alternating 30 s off and 30 s on periods (Dynamic state). Subsequently, two sqBOLD imaging scans using different echo times (TE of 60 ms and TE of 74 ms) were performed while participants viewed a black screen (Baseline state), and a final sqBOLD imaging with TE of 74 ms was conducted while participants viewed a flickering checkerboard pattern (Activated state). **b** In the patient protocol, pre-contrast T1-weighted (T1W), T2-weighted (T2W), and T2-weighted FLAIR (T2W-FLAIR) scans were acquired. This was followed by a sqBOLD scan (TE of 74 ms), GBCA injection, and a post-contrast T1W scan. The duration for each sequence is indicated at the bottom of the respective sections. *sqBOLD* stands for streamlined quantitative BOLD; *GBCA* stands for Gadolinium-Based Contrast Agent

Scanning was performed using a 3 Tesla (Discovery MR750, GE, Waukesha, USA) whole-body MRI scanner equipped with a 32-channel head coil. The MRI protocol for healthy participants is illustrated in Fig. 1a. First, a high-resolution 3D T1-weighted (T1W) scan was acquired at rest for tissue segmentation and registration purposes. The acquisition parameters were TE: 2.1 ms, TR: 6.1 ms, TI: 450 ms, flip angle: 12º, voxel size: 1.0 × 1.0 × 1.0 mm^3^ with whole brain coverage.

The healthy participants, only, underwent visual stimulation to assess the sensitivity of the sqBOLD technique to hemodynamic changes. To determine each participant’s areas of activation to visual stimulation, a BOLD fMRI localizer was conducted, selecting voxels with significant BOLD responses during stimulation. Participants were shown a flickering checkerboard pattern (8 Hz), alternating ON and OFF in 30 s blocks, which we refer to as the “dynamic state” in Fig. 1a. BOLD fMRI data were obtained with a T_2_*-weighted echo-planar imaging (EPI) sequence covering the whole brain with acquisition parameters of TE: 35 ms, TR: 3000 ms, flip angle: 75º, voxel size: 1.7 × 1.7 × 4.0 mm^3^, number of slices: 28, slice thickness: 3.0 mm with 1.0 mm gap between slices, number of TRs: 100, with a total scan duration of 5 min.

After the localizer task, participants were instructed to focus on a black screen while undergoing sqBOLD imaging using a FLAIR asymmetric spin echo (FLAIR-ASE) sequence with an EPI readout [8]. By shifting the refocusing pulse toward the excitation pulse by τ/2, the spin-echo was moved away from the echo-time by an amount τ. The acquisition parameters for FLAIR-ASE were TE: 60 ms, TR: 8000 ms, TI: 2000 ms, flip angle: 90º, voxel size: 2.3 × 2.3 × 3.0 mm^3^, the number of slices: 28, slice thickness: 2.0 mm with 1.0 mm gap between slices. FLAIR-ASE was performed with multiple shift values (τ) of 0, 16, 20, 24, 28, 32, 36, 40, 44, and 48 ms, resulting in a total scan duration of 4 min.

To investigate the impact of TE on the sqBOLD-derived parameters, another sqBOLD imaging was acquired, but with TE values of 74 ms, rather than the original TE of 60 ms. Similarly, participants were instructed to focus on a black screen during data acquisition, which we refer to as the baseline state. Both TE of 60 ms and 74 ms are known to provide a maximum signal-to-noise ratio (SNR) based on the simulation results [8]. While the longer TE allows longer ASE offsets to be acquired, potentially improving the accuracy of *R*_2_^'^, it comes at the cost of an increased scan time. Consequently, FLAIR-ASE was executed with shift values (τ) of 0, 16, 20, 24, 28, 32, 36, 40, 44, 48, 52, 56, and 60 ms, leading to a scan duration of 5 min and 12 s.

The final scan for healthy participants involved performing sqBOLD imaging with visual stimulation, where participants viewed a flickering checkerboard pattern (8Hz) that remained on for the entire scan duration of 5 min and 12 s. This state is referred to as the activated state. A TE of 74 ms was used as it allowed for longer shift values (τ) to be included. This scan, along with the FLAIR-ASE scan with a TE of 74 ms in the baseline state, was used to evaluate sqBOLD sensitivity in detecting changes in *R*_2_^'^, DBV, and OEF between the baseline and activated states.

The MRI protocol used for the two patients consisted of pre-contrast T1-weighted (T1W), T2-weighted (T2W), T2-weighted fluid-attenuated inversion recovery (T2W-FLAIR), and one sqBOLD imaging, all performed at rest (Fig. 1b). Another T1W scan was acquired after the injection of gadolinium-based contrast agent (7.5 mmol of GBCA (Gadovist, Bayer, Leverkusen, DE)). Both pre- and post-contrast T1W scans used the same acquisition parameters as in the healthy participant protocol. T2W scan was acquired with acquisition parameters of TE: 107 ms, TR:10,000 ms, voxel size: 0.5 × 0.5 × 3.2 mm^3^, while the T2W-FLAIR scan was acquired with TE:106 ms, TR: 6000 ms, TI: 1890 ms, voxel size: 0.6 × 0.5 × 0.5 mm^3^. sqBOLD imaging was performed using the same FLAIR-ASE sampling scheme and acquisition parameters as those used for healthy participants with a TE of 74 ms.

### Image processing

In the process of collecting FLAIR-ASE data, brain volumes have been gathered at different shift values (τ), each recorded as a separate volume sequentially in time. These volumes were motion-corrected using the FSL linear motion correction tool (MCFLIRT) [15]. Then, the brain tissue was extracted from the MRI images using the FSL brain extraction tool (BET) on the first volume of the acquisition FLAIR-ASE (τ = 0 ms) [16], with a fractional intensity threshold of 0.2 [16]. The created brain mask was then applied to the computation of sqBOLD-derived maps, exclusively targeting the voxels located within the confines of the brain mask.

**Fig. 2:**
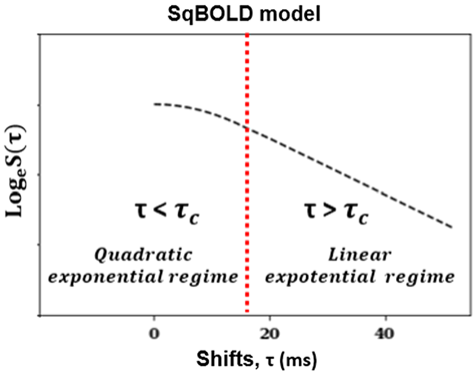
This diagram represents the sqBOLD model, which depicts the logarithmic decay of the MR signal with *τ* shifts. *τ*_c_, set at 16 ms, marks the transition from the quadratic exponential regime to the linear exponential one

*R*_2_^'^, DBV, and OEF maps were estimated from the pre-processed FLAIR-ASE data using the Quantiphyse tool (version 0.9.9) [17]. In every voxel, the MRI signal experiences increased decay as the shift values (τ) increase, i.e., as the readout echo gets farther from the spin-echo. In the sqBOLD technique, the signal decay observed across different shift values (τ) exhibits two distinct patterns, primarily due to the presence of multiple compartments within each voxel of the brain maps. These behaviors are characterized as a quadratic exponential decay for τ < τc and a linear exponential decay for τ > τc (Fig. 2) [18]. τ_c_ is the boundary between these regimes and a conservative estimate of this time is τ_c_ = 16 ms [19]. The signal decay is described by the following equation:

1 $$S\left(\tau \right)=\left\{\begin{array}{c}{S}_{0}exp\left(-{R}_{2}\cdot TE\right)\cdot exp\left(-0.3\cdot \frac{{({R}_{2}^{\prime}\cdot \tau )}^{2}}{DBV}\right) \tau <{\tau }_{c}\\ {S}_{0} exp\left(-{R}_{2}\cdot TE\right)\cdot exp\left(DBV-{R}_{2}^{\prime}\cdot \tau \right) \tau >{\tau }_{c}\end{array}\right.,$$

where the factor *S*_0_ controls for constant terms, *R*_2_ is the irreversible transverse relaxation rate and TE is the echo time; these ca be substituted with *S*(0):

$$\tau =0 \stackrel{yields}{\to } S\left(0\right)={S}_{0}exp\left(-{R }_{2}\cdot \mathrm{TE}\right)$$

To estimate *R*_2_^'^ and DBV from Eq. (1), Quantiphyse uses a non-linear model implemented in a Bayesian framework to fit *R*_2_^'^ and DBV to the pre-processed sqBOLD data [17]. OEF then can be measured by the following equation:

2 $$\mathrm{OEF}= \frac{{3.R}_{2}^{\prime}}{4.\pi .\gamma {B}_{0}{\Delta \chi }_{0}.Hct.DBV}$$

where $\gamma$ is the proton gyromagnetic ratio of 267.5 × 10^6^ rad T^–1^ s^–1^, *B*_0_ is the field strength (3T in this study), ${\Delta \chi }_{0}$ is the susceptibility difference between fully oxygenated and fully deoxygenated red blood cells (0.264 ppm), and Hct is blood hematocrit assumed to be 0.4 [20].

The functional localizer data were pre-processed and analyzed using FEAT v6.00 from FMRIB's Software Library (FSL) [21]. Pre-processing included motion correction, spatial smoothing, and high-pass temporal filtering. Afterward, the data were subjected to general linear model (GLM) analysis, convolving the task design matrix with a canonical hemodynamic response function (HRF) to model the expected BOLD response to visual stimulation. A z-statistics (zstat) map was generated by calculating the statistical significance of the BOLD response at each voxel using a *t* test. A volume of interest (VOI), which we refer to as the fMRI-based VOI, was created using a threshold of *Z* > 3.1 and a corrected cluster significance threshold of *P* = 0.05.

Several VOIs were used for analysis in the healthy participants, comprising the gray matter (GM), visual cortex (VC), non-visual cortex (non-VC), the fMRI-based VOI, and a non-fMRI-based VOI. The GM VOI was generated using the FAST segmentation tool in FSL, which clusters the T1W image into three distinct regions: GM, WM, and CSF. A partial volume threshold of 0.9 was employed to ensure accurate delineation of GM regions [22]. The visual cortex (VC) VOI was selected from the “Juelich Histological Atlas” and transferred to the individual T1W space using the Elastix registration tool (https://elastix.lumc.nl/) [23]. To achieve this, the T1W image from MNI152 standard space was registered non-rigidly to the individual T1W space to determine the transformation matrix. Then, the transformation matrix was applied to transfer the VC VOI from the atlas to the individual T1W space. GM and VC VOIs were transferred rigidly from T1W space to the first volume of FLAIR-ASE (*τ* = 0 ms) with the Elastix registration tool. Finally, the fMRI-based VOI was binarized and transformed to the FLAIR-ASE (*τ* = 0 ms) using the same transformation matrix generated by rigid registration (with Elastix) between the first volume of the fMRI and the first volume of FLAIR-ASE (*τ* = 0 ms). The sequence of steps detailing the processing of FLAIR-ASE, the generation of VOIs, and their subsequent registration to FLAIR-ASE (*τ* = 0 ms), is visually represented in Supplementary Fig. 1.

It should be emphasized that all analyses only considered GM voxels within the fMRI-based VOI and VC VOI. In other words, when we refer to the fMRI-based VOI and VC VOI, we specifically mean the GM voxels within these regions. Lastly, in order to assess if the observed changes in the sqBOLD parameters were a consequence of the visual task or some other factors, we also inspected alterations in the sqBOLD parameters within "control" areas. These areas included GM voxels located away from the visual cortex and fMRI-based VOIs, referred to as non-VC VOI and non-fMRI-based VOI, respectively.

### Statistical analysis

*R*_2_^'^, DBV, and OEF maps were acquired for healthy participants for each sqBOLD session separately. Median values of the *R*_2_^'^, DBV, and OEF were measured for various VOIs in each healthy participant individually. A group-level analysis was carried out solely for healthy participants, where the calculated *R*_2_^'^, DBV, and OEF in GM voxels were compared across individuals. The goal of this comparison was to assess FLAIR-ASE results with TE of 60 ms and TE of 74 ms. Furthermore, a group-level analysis was conducted for the calculated *R*_2_^'^, DBV, and OEF in both baseline and activated states (TE of 74 ms), aiming to investigate the sensitivity of these sequences in detecting changes in these parameters.

Group comparisons were performed using paired two-sided t-tests or Wilcoxon signed-rank tests, depending on the Shapiro–Wilk test for normality outcomes. If the Shapiro–Wilk test indicated that the differences between paired observations followed a normal distribution, a paired two-sided *t* test was employed. Otherwise, a Wilcoxon signed-rank test was utilized as a nonparametric alternative. A *p* value of 0.05 or lower was considered statistically significant for all tests. Furthermore, *R*_2_^'^, DBV, and OEF maps were acquired for two patients and were visually assessed by an experienced radiologist with regard to the overall quality of the images and the ability to identify pathologies. This evaluation was conducted to illustrate the potential utility of this technique in a clinical setting.

## Results

### sqBOLD in two TEs

**Fig. 3:**
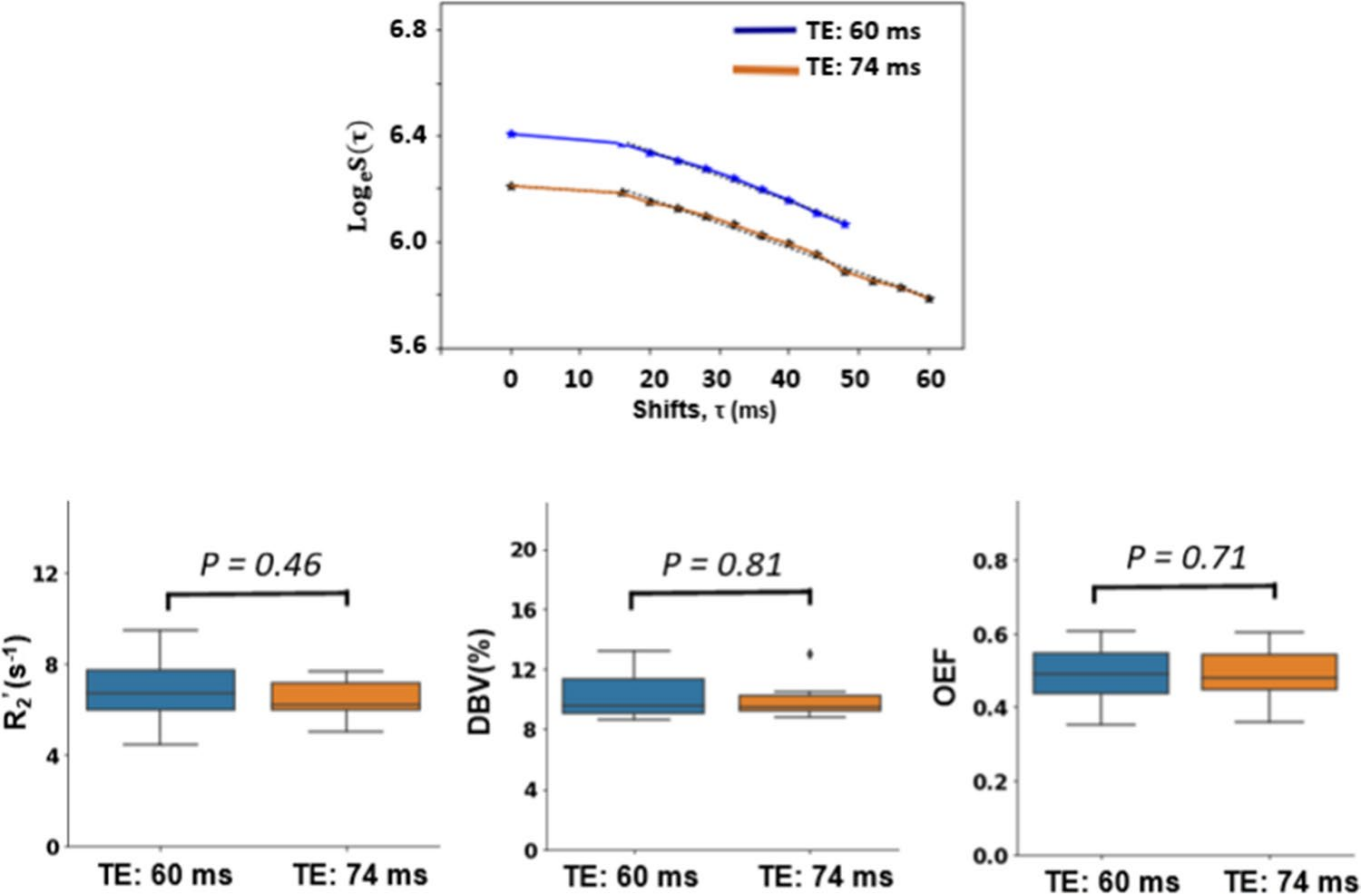
Top row: the average logarithm of sqBOLD signals in gray matter voxels (gray matter partial volume estimate > 0.9) across the whole brain at two echo times (TE of 60 ms and 74 ms in the baseline state) for a healthy participant. Bottom row: Boxplots presenting the group statistics of *R*_2_^'^, DBV, and OEF measurements, comparing two echo times (TE of 60 ms and 74 ms in the baseline state) in gray matter voxels for healthy participants

The average of the logarithm of recorded signals in GM voxels for one randomly selected healthy participant has been shown in Fig. 3. As expected, the signals decrease as the shift values (*τ*) increases, following the same expected patterns depicted in Fig. 2. The signal obtained with a TE of 60 ms displayed a higher signal offset compared to that of TE of 74 ms. In contrast, using a TE of 74 ms enabled the inclusion of more shifts in the measurements compared to using a TE of 60 ms. Nonetheless, as shown in the boxplot in the lower row, no significant differences in the group-averaged *R*_2_^'^, DBV, and OEF were observed with the two TEs.

### sqBOLD in baseline vs activated states

To assess changes in the sqBOLD-derived parameters due to functional activation, a BOLD fMRI localizer was first run to create a functionally defined VOI (fMRI-based VOI). A structurally defined visual cortex VOI (VC VOI) was also used. The localizer experiments consistently demonstrated robust activation in the visual cortex areas for each individual participant, and for some participants, also exhibited activation in specific deep gray matter structures involved in visual processing (see Supplementary Fig. 1).

**Fig. 4:**
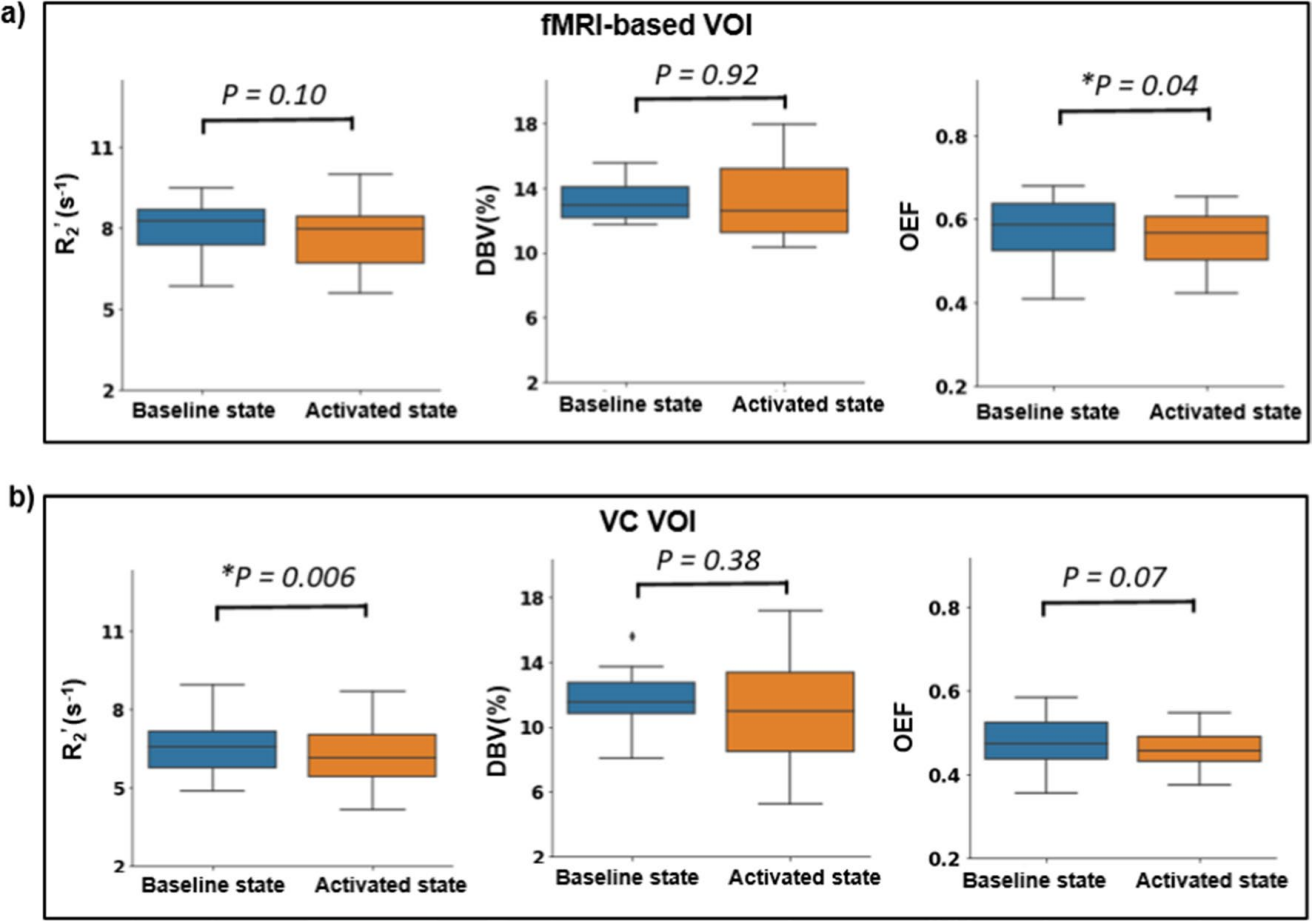
Boxplots presenting the group averages of *R*_2_^'^, DBV and OEF measurements, comparing two states: baseline and activated in gray matter voxels located in **a** fMRI-based VOI and **b** visual cortex (VC) for healthy participants. Statistically significant results (*P* < 0.05) are denoted with *

Figure 4 presents the results for the group comparison of the sqBOLD-derived parameters collected in the baseline and the activated states (both with a TE of 74 ms). Since the Shapiro–Wilk test for normality was not rejected in these evaluations, a paired two-sided *t* test was utilized for the comparisons. The group-averaged (*N* = 8) OEF decreased from 0.56 ± 0.09 in the baseline state to 0.54 ± 0.07 in the activated state (*p* = 0.04) in the fMRI-based VOI, while the decrease of OEF from 0.47 ± 0.07 in the baseline state to 0.45 ± 0.05 in the activated state was not significant (*p* = 0.07) in the VC VOI. *R*_2_^'^ showed no statistically significant difference between baseline 7.9 ± 1.2 s^−1^ and stimulation 7.7 ± 1.4 s^−1^ (*p* = 0.10) in the fMRI-based VOI, but in the VC VOI a significant decrease was observed in *R*_2_^'^, from 6.5 ± 1.3 s^−1^ in the baseline state to 6.2 ± 1.4 s^−1^ in the activated state (*p* = 0.006). DBV did not show a consistent nor significant change in any of these VOIs (Fig. 4). Supplementary Fig. 2 illustrates the changes in *R*_2_^'^, DBV, and OEF at the individual level at both baseline and activated states. The majority of participants showed a similar trend of reduction in *R*_2_^'^ and OEF from baseline to stimulation, while DBV changes varied across individuals. Conversely, Supplementary Fig. 3 shows that the sqBOLD-derived measurements in the non-fMRI-based VOI and the non-VC VOI showed no consistent nor significant change between the two states in *R*_2_^'^, DBV, and OEF. This suggests that the observed changes in the sqBOLD parameters in the fMRI-based and VC VOIs were, indeed, due to physiological changes induced by the visual task.

### sqBOLD in the patients

**Fig. 5:**
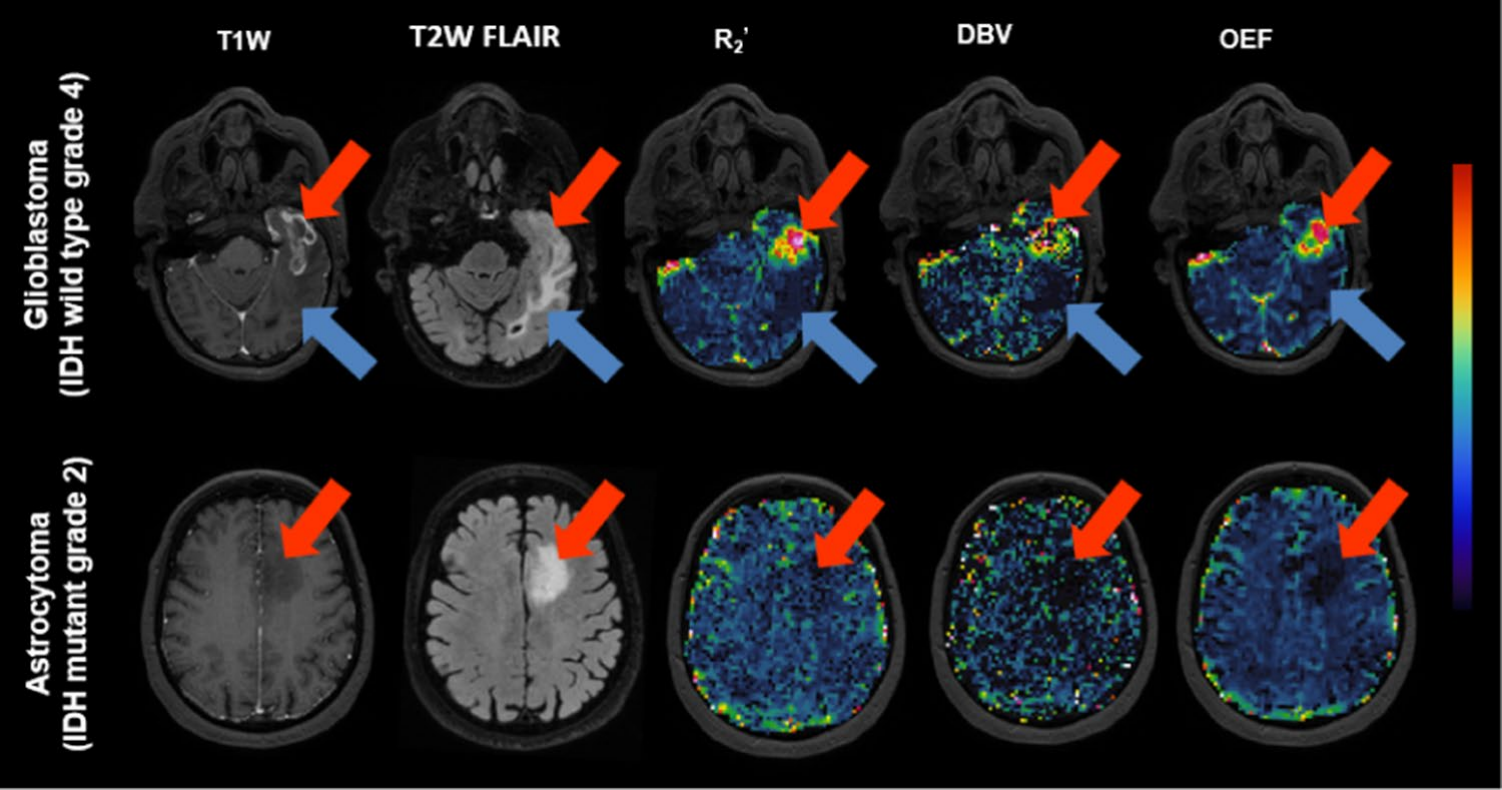
Example slices of two patients with glioblastoma (grade 4) and astrocytoma (grade 2). The images from left to right are, respectively: post-contrast T1W, T2W FLAIR, *R*_2_^'^ (0–40 ${\mathrm{s}}^{-1}$), DBV (0–10%) and OEF (0–1). Arrows indicate different parts of the tumor: in the first patient, the red arrow points to the contrast enhancing area and necrotic core of the tumor, while the blue arrow points to the surrounding edema. In the second patient, the red arrow points to the tumor area

*R*_2_^'^, DBV, and OEF maps were obtained and visually assessed for two patients: one with high-grade glioma (glioblastoma, IDH wild type grade 4) and another with low-grade glioma (astrocytoma, IDH mutant grade 2). Figure 5 demonstrates that in the first patient, *R*_2_^'^, DBV, and OEF values in areas marked by red arrows—including the enhancing ring of the tumor and the necrotic core—exhibit higher values compared to healthy brain tissue, albeit with a heterogeneous pattern. Additionally, for this patient, the edema area surrounding the enhancing region (indicated by blue arrows) displays lower values in *R*_2_^'^, DBV, and OEF compared to the rest of the brain. In contrast, the non-enhancing tumor (indicated by red arrows) in the second patient shows lower values in *R*_2_^'^, DBV, and OEF compared to the rest of the brain.

## Discussion

The current study evaluated the ability of sqBOLD to measure changes in brain oxygen-related parameters in response to visual stimulation in healthy participants. The results demonstrated a statistically significant decrease in oxygen extraction fraction (OEF) by 3.5% and reversible transverse relaxation rate (*R*_2_^'^) by 4.6% in a volume of interest defined by a functional localizer and the visual cortex, respectively. Furthermore, the study demonstrated the potential of sqBOLD as a clinically feasible (< 6 min) and non-invasive technique for measuring brain oxygen-related parameters in patients with brain tumors. The visual assessment of sqBOLD-derived maps in two patients revealed a heterogeneous pattern of parameters both within and between patients.

We first investigated the effect of TE on our local implementation of the sqBOLD acquisition. Based on visual inspection of raw FLAIR-ASE images, our results suggest that the data obtained using both TE values have a similarly good signal-to-noise ratio (SNR), which is consistent with previous studies that have confirmed this using simulated data [9]. Our results showed that no significant difference was found between using TE of 74 ms and 60 ms in estimating *R*_2_^'^, DBV and OEF. We chose to move forward with a TE of 74 ms for our sqBOLD acquisition as it allowed incorporating longer τ, which potentially permits improved accuracy in *R*_2_^'^ estimation for regions with low *R*_2_^'^, and sensitivity to subtle changes in *R*_2_^'^.

sqBOLD-derived parameters were measured for gray matter (GM) voxels in healthy participants, revealing higher *R*_2_^'^ values in these VOIs than in previous studies [5]. One possible culprit could be that the 3 mm slice thickness used during data collection may have led to *R*_2_^'^ overestimation in larger voxels, due to intra-voxel dephasing caused by macroscopic magnetic field inhomogeneity at tissue interfaces. While a multi-slab acquisition could potentially reduce this effect, there is a trade-off in brain coverage and scan time, and we opted for a more reasonable scan time using 2D multi-slice imaging in this study to balance these factors [9, 24]. Consequently, higher *R*_2_^'^ values can lead to high OEF values (0.52 on average) above the expected range in healthy participants (0.3–0.5) [25].

We then performed an assessment of the sqBOLD technique's capability to detect changes in oxygen-related parameters under two conditions: with and without visual stimulation. The group-averaged *R*_2_^'^ and OEF values decreased in the visual cortex (VC) and fMRI-based VOIs in response to visual stimulation, but the decrease was only statistically significant in the VC for *R*_2_^’^ and in the fMRI-based VOI for OEF. These changes are consistent with typical neurovascular coupling, where increased neuronal activity results in increased blood flow, causing an overshoot in oxygenated blood delivery and a decrease of the deoxyhaemoglobin concentration in the activated area [26]. As a result, *R*_2_^'^, which is sensitive to the magnetic field inhomogeneity between deoxygenated blood and the surrounding tissue, decreases [27], leading to a reduction in the OEF.

We are aware of only one study that had previously measured *R*_2_^'^ during visual stimulation, reporting a drop of 4.3% in the fMRI-activated area using multi-parametric quantitative BOLD (mq-BOLD) technique [28]. This value is more or less consistent with the 4.6% decrease in *R*_2_^'^ in the VC in our study. In contrast to *R*_2_^'^, several studies have investigated OEF changes during visual stimulation. Our study found a 3.5% decrease in OEF in the fMRI-based VOI. However, other studies have reported inconsistent values, such as 6.2% in a study by Wise et al., 20.6% in a study by Gauthier et al. (both using calibrated fMRI), and 18.6% in a study by Stout et al. [29–31]. Yin et al. reported average relative negative changes in OEF of 11.8% under normoxia conditions in motor task studies [32]. None of the mentioned studies used the sqBOLD technique to measure OEF, which hampers direct comparisons with our results. However, the smaller changes in OEF observed in our study may be attributed to the continuous visual stimulation applied during the sqBOLD data collection. In fact, participants were exposed to a flickering checkerboard screen for 5 min and 12 s during the activated state and 5 min and 12 s of black screen during the baseline to allow time for running FLAIR-ASE sequence. Extended visual stimulation during sqBOLD data collection might result in neuronal habituation, possibly leading to a decrease in hemodynamic responses and reduced sensitivity in detecting OEF changes between states [29].

In comparing the responses from the fMRI-based VOIs and the VC VOI, the fMRI-based VOIs were mostly larger than the anatomically-defined VC VOI (supplementary Fig. 1), and in some participants, the fMRI-based VOI covered areas with high iron content such as the deep gray matter [33]. High iron content in the tissue could potentially contribute to increased magnetic field inhomogeneities, leading to the overestimation of sqBOLD-derived parameters. As shown in Fig. 4, *R*_2_^'^, DBV, and OEF were higher in the fMRI-based VOI compared to the anatomically-defined VC VOI, indicating a possible influence of iron content on the measurements. Considering the small sample size, the fairly lenient threshold of 3.1 in the fMRI analysis, and the use of spatial smoothing, each may have implications on the results, highlighting the need for cautious interpretation. However, the non-significant changes in *R*_2_^'^ and OEF in non-fMRI or non-VC-based VOIs during the visual stimulation suggest that the significant changes observed in the fMRI-based and VC VOIs were likely physiologically driven rather than measurement-related errors (supplementary Fig. 3).

In addition to task-induced sqBOLD parameter changes, this study assessed the feasibility of the sqBOLD technique for clinical application on two patients with different tumor grades. The generated brain maps of *R*_2_^'^, DBV, and OEF displayed diagnostic quality in a sensible range, suggesting potential utility in clinical settings. Visual inspection of these maps revealed increased intensity heterogeneity in contrast-enhanced areas and the necrotic core of high-grade glioblastoma compared to contralateral healthy tissue, while decreased intensity was uniformly observed in the edema area around the tumor in high-grade as well as the tumor area in low-grade astrocytoma. These observations are consistent with previous studies examining OEF in gliomas using alternative methodologies, suggesting a correlation between heightened OEF and tumor aggressiveness [7, 8, 12, 34].

A limitation of this study is the lack of cerebral blood flow (CBF) measurements. To provide a comprehensive understanding of oxygen metabolism, CBF should be measured alongside OEF. In healthy participants, we assumed that CBF increases in the stimulated area [9]. However, in tumors, CBF is rather inconsistent across various tumor regions and needs to be measured separately using clinically well-established MRI techniques such as arterial spin labeling (ASL) or dynamic susceptibility contrast (DSC). Furthermore, the study assumes a fixed hematocrit value of 0.4, based on a mean population value, despite known variation among subjects. Underestimating hematocrit causes an underestimation of oxygenated blood supply to the area and subsequent OEF overestimation.

We chose to focus our analysis solely on gray matter voxels located in the desired VOIs and excluded signal from WM for two reasons: first, highly oriented myelin sheaths surrounding axons can result in axon fiber orientation-dependent changes in *T*_2_^*^, independent of DBV and OEF, therefore biasing the qBOLD estimates [35]. Second, a key assumption of the qBOLD model is that the blood vessels within the voxel are all randomly oriented. This is, generally, not the case in white matter, where a significant fraction of the blood vessels run parallel to the fiber bundle orientations [36]. Therefore, the quantitative analysis of qBOLD-derived parameters in white matter may result in inaccurate OEF estimation using qBOLD techniques without the implementation of additional methods or modifications [37].

Another limitation is that the qBOLD technique is known for frequently overestimating DBV, including in our own study. This overestimation primarily occurs because the qBOLD model assumes that protons are in the static dephasing regime. However, in reality, the presence of diffusion in tissue leads to imperfect spin-echo refocusing and, therefore, a potentially larger mismatch between the measured spin-echo signal and the extrapolated one, resulting in the observed overestimation of DBV [38]. This overestimation, in turn, affects the accuracy of the measured OEF due to the mutual reliance of *R*_2_^'^ on DBV and OEF, as expressed by Eq. (2) [39]. Some studies involving glioma patients have addressed this problem by employing the relative total cerebral blood volume (rCBV) derived from a dynamic susceptibility contrast scan instead of DBV [8]. This solution was not adopted in the present study due to the absence of contrast injection in the healthy participants' protocol, but it will be taken into consideration for future patient studies.

To ensure clinical applicability and uniform implementation across different centers with minimal variability and high repeatability, two key steps can be taken. First, in the acquisition phase, it is important to harmonize the acquisition protocol by implementing a standardized approach. Conducting a traveling head study can help evaluate inter-site variability and allow for necessary adjustments to achieve consistent results. Second, in the analysis phase, utilizing open-source software such as Quantiphyse can be beneficial. This software enables the processing of collected data to generate sqBOLD-derived maps, ensuring consistency and facilitating result comparison across various centers. These steps enhance the clinical utility and reliability of the findings.

### Conclusion

The findings of this study suggest that sqBOLD can be a promising non-invasive tool for quantitatively studying changes in brain physiology in response to stimuli in healthy subjects. Additionally, the sqBOLD technique demonstrated potential utility in clinical settings for assessing brain tumor oxygenation. The non-invasive and quantitative nature of this method makes it well-suited for clinical applications, longitudinal monitoring of patients, and evaluating treatment and intervention outcomes. Future research will emphasize applying the technique to a larger patient cohort, integrating perfusion measurements, and addressing the aforementioned challenges of sqBOLD in the context of brain pathologies.

### Supplementary Information

Below is the link to the electronic supplementary material.

Supplementary Fig. 1A detailed illustration of the processing pipeline. The topmost row outlines the steps involved in the processing of FLAIR-ASE data. The subsequent rows, in descending order, detail the generation and registration of gray matter (GM), visual cortex (VC), and fMRI-based Volumes of Interest (VOIs), to FLAIR-ASE (τ = 0 ms), respectively. (JPG 112 KB)

Supplementary Fig. 2 Scatter plots showing the median of *R*_2_^’^, DBV, and OEF in gray matter voxels located in a) fMRI-based VOI and b) visual cortex (VC) for healthy participants. Individual participants' data are connected by colored lines, with one end representing the values in the baseline state and the other end indicating the values in the activated state. (JPG 53 KB)

Supplementary Fig. 3 Boxplots presenting the group averages of *R*_2_^'^, DBV and OEF measurements, comparing two states: baseline and activated in gray matter voxels located in a) non-fMRI-based VOI and b) non-visual cortex (VC) for healthy participants. Statistically significant results (P < 0.05) are denoted with *. (JPG 49 KB)

## Data Availability

Data are available on request.

## References

[1] Muir ER, Cardenas DP, Duong TQ (2016). MRI of brain tissue oxygen tension under hyperbaric conditions. Neuroimage.

[2] Jensen RL (2009). Brain tumor hypoxia: tumorigenesis, angiogenesis, imaging, pseudoprogression, and as a therapeutic target. J Neuro-Oncol.

[3] He X, Yablonskiy DA (2007). Quantitative BOLD: mapping of human cerebral deoxygenated blood volume and oxygen extraction fraction: default state. Magn Reson Med.

[4] Pauling L, Coryell CD (1936). The magnetic properties and structure of hemoglobin, oxyhemoglobin and carbonmonoxyhemoglobin. Proc Natl Acad Sci.

[5] Ni W, Christen T, Zun Z, Zaharchuk G (2015). Comparison of R2′ measurement methods in the normal brain at 3 tesla. Magn Reson Med.

[6] Blockley NP, Griffeth VEM, Simon AB, Dubowitz DJ, Buxton RB (2015). Calibrating the BOLD response without administering gases: Comparison of hypercapnia calibration with calibration using an asymmetric spin echo. Neuroimage.

[7] Yao J, Chakhoyan A, Nathanson DA, Yong WH, Salamon N, Raymond C, Mareninov S, Lai A, Nghiemphu PL, Prins RM, Pope WB, Everson RG, Liau LM, Cloughesy TF, Ellingson BM (2019). Metabolic characterization of human IDH mutant and wild type gliomas using simultaneous pH- and oxygen-sensitive molecular MRI. Neuro Oncol..

[8] Tóth V, Förschler A, Hirsch NM, Den Hollander J, Kooijman H, Gempt J (2013). MR-based hypoxia measures in human glioma. J Neurooncol.

[9] Berman AJL, Mazerolle EL, MacDonald ME, Blockley NP, Luh WM, Pike GB (2018). Gas-free calibrated fMRI with a correction for vessel-size sensitivity. Neuroimage.

[10] Stone AJ, Harston GWJ, Carone D, Okell TW, Kennedy J, Blockley NP (2019). Prospects for investigating brain oxygenation in acute stroke: experience with a non-contrast quantitative BOLD based approach. Hum Brain Mapp.

[11] Seiyama A, Seki J, Tanabe HC, Sase I, Takatsuki A, Miyauchi S (2004). Circulatory basis of fMRI signals: relationship between changes in the hemodynamic parameters and BOLD signal intensity. Neuroimage.

[12] Preibisch C, Shi K, Kluge A, Lukas M, Wiestler B, Göttler J (2017). Characterizing hypoxia in human glioma: a simultaneous multimodal MRI and PET study. NMR Biomed.

[13] Stadlbauer A, Mouridsen K, Doerfler A, Bo Hansen M, Oberndorfer S, Zimmermann M (2018). Recurrence of glioblastoma is associated with elevated microvascular transit time heterogeneity and increased hypoxia. J Cereb Blood Flow Metab.

[14] Louis DN, Perry A, Wesseling P, Brat DJ, Cree IA, Figarella-Branger D (2021). The 2021 WHO Classification of Tumors of the Central Nervous System: a summary. Neuro Oncol.

[15] Jenkinson M (2002). Improved Optimization for the Robust and Accurate Linear Registration and Motion Correction of Brain Images. Neuroimage.

[16] Smith SM (2002). Fast robust automated brain extraction. Hum Brain Mapp.

[17] Cherukara MT, Stone AJ, Chappell MA, Blockley NP (2019). Model-based Bayesian inference of brain oxygenation using quantitative BOLD. Neuroimage.

[18] Yablonskiy DA, Haacke EM (1994). Theory of NMR signal behavior in magnetically inhomogeneous tissues: The static dephasing regime. Magn Reson Med.

[19] Blockley NP, Stone AJ (2016). Improving the specificity of R2’ to the deoxyhaemoglobin content of brain tissue: prospective correction of macroscopic magnetic field gradients. Neuroimage.

[20] Spees WM, Yablonskiy DA, Oswood MC, Ackerman JJH (2001). Water proton MR properties of human blood at 15 Tesla: magnetic susceptibility, T1, T2, T*2, and non-Lorentzian signal behavior. Magn Reson Med.

[21] Woolrich MW, Behrens TEJ, Beckmann CF, Jenkinson M, Smith SM (2004). Multilevel linear modelling for FMRI group analysis using Bayesian inference. Neuroimage.

[22] Zhang Y, Brady M, Smith S (2001). Segmentation of brain MR images through a hidden Markov random field model and the expectation-maximization algorithm. IEEE Trans Med Imaging.

[23] Klein S, Staring M, Murphy K, Viergever MA, Pluim J (2010). elastix: a toolbox for intensity-based medical image registration. IEEE Trans Med Imaging.

[24] Blockley NP, Griffeth VEM, Stone AJ, Hare HV, Bulte DP (2015). Sources of systematic error in calibrated BOLD based mapping of baseline oxygen extraction fraction. Neuroimage.

[25] Raichle ME, MacLeod AM, Snyder AZ, Powers WJ, Gusnard DA, Shulman GL (2001). A default mode of brain function. Proc Natl Acad Sci.

[26] Donahue MJ, Hoogduin H, van Zijl PCM, Jezzard P, Luijten PR, Hendrikse J (2011). Blood oxygenation level-dependent (BOLD) total and extravascular signal changes and Δ R 2 * in human visual cortex at 1.5, 3.0 and 7.0 T. NMR Biomed.

[27] Fujita N, Matsumoto K, Tanaka H, Watanabe Y, Murase K (2006). Quantitative study of changes in oxidative metabolism during visual stimulation using absolute relaxation rates. NMR Biomed.

[28] Preibisch C, Koutsouli S, Kaczmarz S, Epp S, Riedl, V (2019) Quantitative functional imaging of visual cortex activity in humans using multi-parametric blood oxygenation level dependent MRI. In: Proceedings of the 27th annual meeting ISMRM. Retrieved from https://cds.ismrm.org/protected/19MProceedings/PDFfiles/3736.html

[29] Wise RG, Harris AD, Stone AJ, Murphy K (2013). Measurement of OEF and absolute CMRO2: MRI-based methods using interleaved and combined hypercapnia and hyperoxia. Neuroimage.

[30] Gauthier CJ, Desjardins-Crépeau L, Madjar C, Bherer L, Hoge RD (2012). Absolute quantification of resting oxygen metabolism and metabolic reactivity during functional activation using QUO2 MRI. Neuroimage.

[31] Stout JN, Adalsteinsson E, Rosen BR, Bolar DS (2018). Functional oxygen extraction fraction (OEF) imaging with turbo gradient spin echo QUIXOTIC (Turbo QUIXOTIC). Magn Reson Med.

[32] Yin Y, Shu S, Qin L, Shan Y, Gao JH, Lu J (2021). Effects of mild hypoxia on oxygen extraction fraction responses to brain stimulation. J Cereb Blood Flow Metab.

[33] Li Y, Sethi SK, Zhang C, Miao Y, Yerramsetty KK, Palutla VK (2021). Iron content in deep gray matter as a function of age using quantitative susceptibility mapping: a multicenter study. Front Neurosci.

[34] Jiao D, Cai Z, Choksi S, Ma D, Choe M, Kwon HJ (2018). Necroptosis of tumor cells leads to tumor necrosis and promotes tumor metastasis. Cell Res.

[35] Sati P, Silva AC, van Gelderen P, Gaitan MI, Wohler JE, Jacobson S (2012). In vivo quantification of T2⁎ anisotropy in white matter fibers in marmoset monkeys. Neuroimage.

[36] Hernández-Torres E, Kassner N, Forkert ND, Wei L, Wiggermann V, Daemen M (2017). Anisotropic cerebral vascular architecture causes orientation dependency in cerebral blood flow and volume measured with dynamic susceptibility contrast magnetic resonance imaging. J Cereb Blood Flow Metab.

[37] Kaczmarz S, Göttler J, Zimmer C, Hyder F, Preibisch C (2020). Characterizing white matter fiber orientation effects on multi-parametric quantitative BOLD assessment of oxygen extraction fraction. J Cereb Blood Flow Metab.

[38] Dickson JD, Ash TWJ, Williams GB, Harding SG, Carpenter TA, Menon DK (2010). Quantitative BOLD: the effect of diffusion. J Magn Reson Imaging.

[39] Sedlacik J, Reichenbach JR (2010). Validation of quantitative estimation of tissue oxygen extraction fraction and deoxygenated blood volume fraction in phantom and in vivo experiments by using MRI. Magn Reson Med.

