# Supplementary figures and images for: Streamlined quantitative BOLD for detecting visual stimulus-induced changes in oxygen extraction fraction in healthy participants: toward clinical application in human glioma
Source: MAGMA. 2023 Aug 9;36(6):975–84. doi: 10.1007/s10334-023-01110-1 (PMC10667381; doi:10.1007/s10334-023-01110-1)

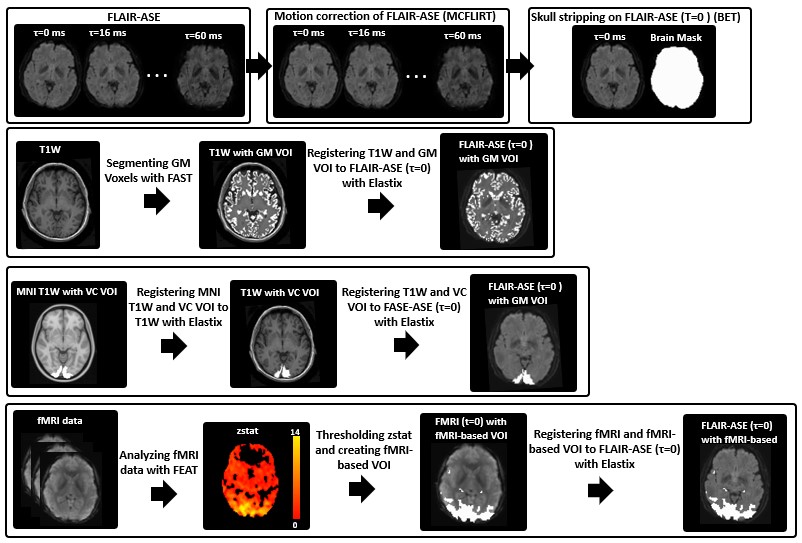

Supplement: Supplementary file 1 — Supplementary Fig. 1A detailed illustration of the processing pipeline. The topmost row outlines the steps involved in the processing of FLAIR-ASE data. The subsequent rows, in descending order, detail the generation and registration of gray matter (GM), visual cortex (VC), and fMRI-based Volumes of Interest (VOIs), to FLAIR-ASE (τ = 0 ms), respectively. (JPG 112 KB) [file 10334_2023_1110_MOESM1_ESM.jpg]

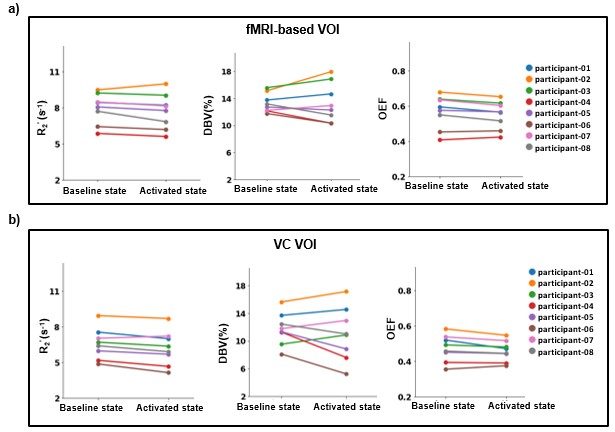

Supplement: Supplementary file 2 — Supplementary Fig. 2 Scatter plots showing the median of R2’, DBV, and OEF in gray matter voxels located in a) fMRI-based VOI and b) visual cortex (VC) for healthy participants. Individual participants' data are connected by colored lines, with one end representing the values in the baseline state and the other end indicating the values in the activated state. (JPG 53 KB) [file 10334_2023_1110_MOESM2_ESM.jpg]

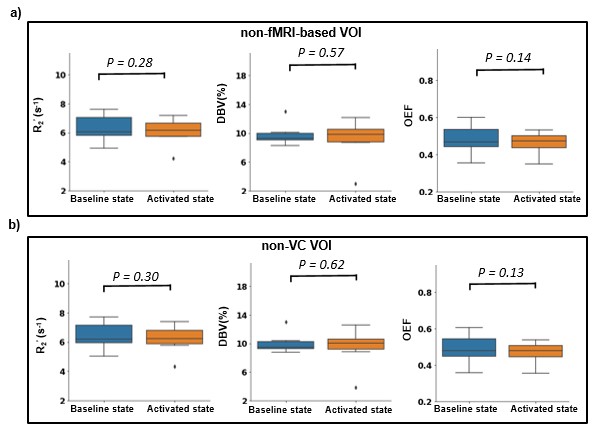

Supplement: Supplementary file 3 — Supplementary Fig. 3 Boxplots presenting the group averages of R2', DBV and OEF measurements, comparing two states: baseline and activated in gray matter voxels located in a) non-fMRI-based VOI and b) non-visual cortex (VC) for healthy participants. Statistically significant results (P < 0.05) are denoted with *. (JPG 49 KB) [file 10334_2023_1110_MOESM3_ESM.jpg]
